# An Old Vulcanite Plate

**Published:** 1884-09

**Authors:** George Watt


### An Old Vulcanite Plate.

BY GEORGE WATT.

Read before the Mad River Valley Dental Society.

In 1856, in the month of August, the American Dental Convention met in Hope Chapel, New York. Amid a group of dentists outside of the chapel, before the meeting was called to order, we found an enthusiastic man, zealously advocating the introduction of a new kind of dental plate, a specimen of which he was wearing. He would take his plate out to show it, put it back to answer questions, snatch it out again, and again return it, almost as fast as one could count the changes. We watched our opportunity, and then and there, amid a brief interview, we got our first sight of a vulcanite dental plate. At the same time we made an arrangement with him to pack and vulcanize a plate for us; and we fitted the teeth, antagonized them with the patient's under ones, sent the case to our newly acquired friend, who made the mold, packed and vulcanized, and sent the denture to us by express. He told us that there were but two dental vulcanizers in America at that time, one in New Haven, and the other in New York; and he stated that ours was probably the first vulcanite plate made for practical use west of the Alleghanies. The plate was worn, and constantly used something over sixteen years. The patient died in 1873, of a putrid form of erysipelas, and the lips were so destroyed that this plate could not be replaced in the mouth by non-professional friends, and no others were present. If a sight of the plate will be of any interest to you as an historical relic, it affords us pleasure to show it to you; simply informing you that it is vulcanized gutta-percha, which as you see, is still in good condition.

